# Supplementary material for: Predictors for one-year outcomes of cardiorespiratory fitness and cardiovascular risk factor control after cardiac rehabilitation in elderly patients: The EU-CaRE study
Source: PLoS One. 2021 Aug 5;16(8):e0255472. doi: 10.1371/journal.pone.0255472 (PMC8341663; doi:10.1371/journal.pone.0255472)
Supplement: S1 Fig — Shown are pooled estimates and 99% confidence intervales (using rubins rule) from the robust linear mixed models over the multiple imputed data (5 imputations with predictive mean matching). CRF at T0, attended number of training sessions, inactivity, lag time to CR start and duration of CR were included a priori. The other variables were significant variables in the model for the pooled data but shown here for the surgery and non-surgery populations separately. Centre was included as random factor. COPD, chronic obstructive pulmonary disease; ACS, acute coronary syndrome; DM, diabetes mellitus; CR, cardiac rehabilitation. (DOCX) [file pone.0255472.s002.docx]

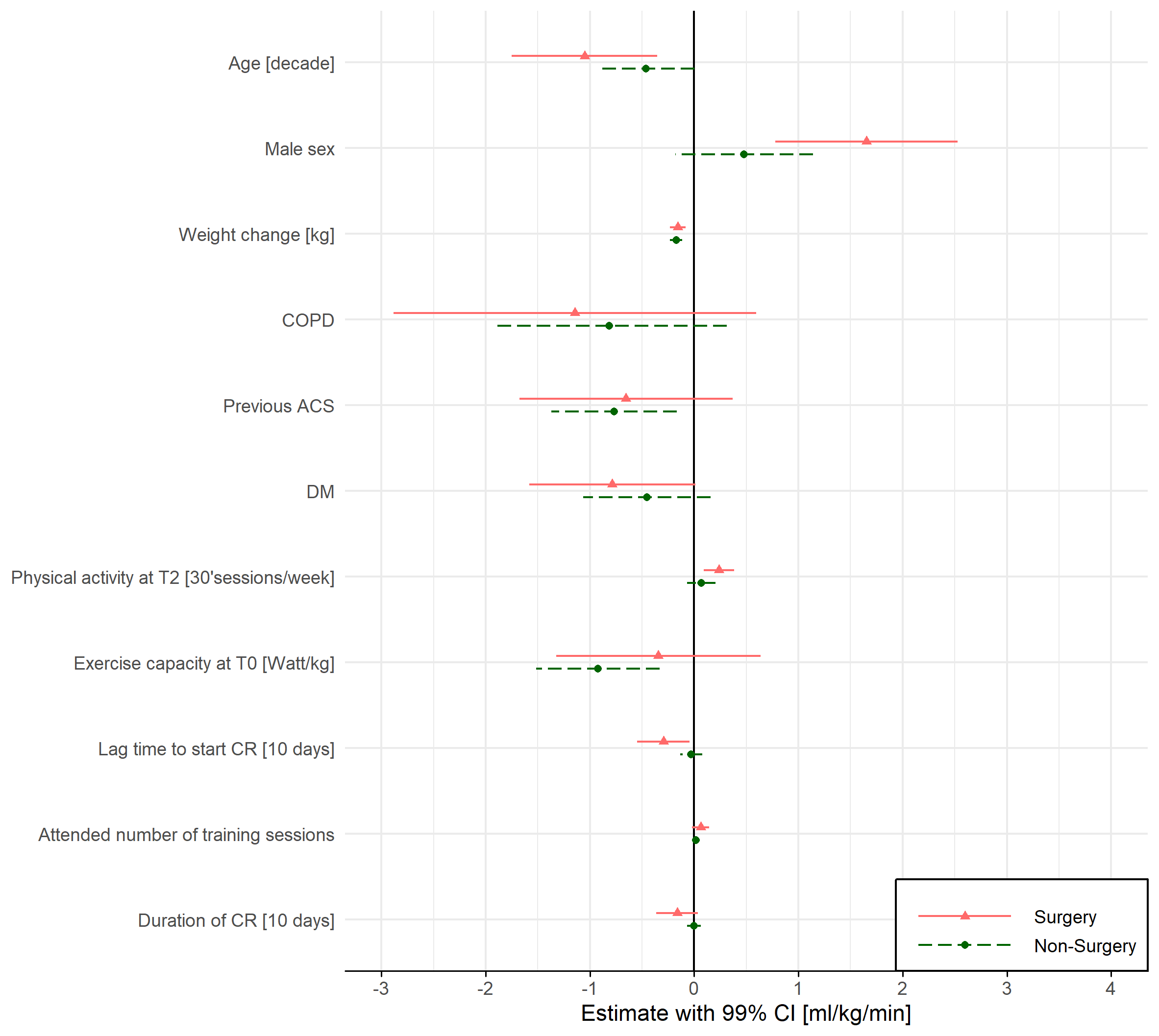


Supplement Figure 1: Predictors for change in peak VO2 for 614 surgery and 1019 non-surgery patients. Shown are pooled estimates and 99% confidence intervales (using rubins rule) from the robust linear mixed models over the multiple imputed data (5 imputations with predictive mean matching). CRF at T0, attended number of training sessions, inactivity, lag time to CR start and duration of CR were included a priori. The other variables were significant variables in the model for the pooled data but shown here for the surgery and non-surgery populations separately. Centre was included as random factor.

COPD, chronic obstructive pulmonary disease; ACS, acute coronary syndrome; DM, diabetes mellitus; CR, cardiac rehabilitation
