# Supplementary material for: Predictors for one-year outcomes of cardiorespiratory fitness and cardiovascular risk factor control after cardiac rehabilitation in elderly patients: The EU-CaRE study
Source: PLoS One. 2021 Aug 5;16(8):e0255472. doi: 10.1371/journal.pone.0255472 (PMC8341663; doi:10.1371/journal.pone.0255472)
Supplement: S1 Table — (DOCX) [file pone.0255472.s003.docx]

The following variables were entered into robust linear models:

Patient anthropometric data: Age, height, BMI, waist circumference, sex, blood pressure;

Socio-behavioural factors: Education, volunteer work, form of living;

Index procedure: CABG, PCI, VHD, stable CAD without revascularization;

Cardiovascular risk factors: Diabetes mellitus, smoking, hypertension, hypocholesteraemia, family CVD history, alcohol consumption, physical inactivity;

Exercise limiting medication: Beta blocker;

Comorbidities: Nephropathy, rheumatic disease, chronic obstructive pulmonary disease, peripheral arterial disease, depression, obstructive sleep apnoea, cerebrovascular accident;

Cardiac complaints: Angina pectoris, chronic heart failure, arterial fibrillations, rhythm other than sinus, other complaints;

Cardiac disease history: Previous CABG, PCI or ACS;

Resting heart and lung function: Forced vital capacity, forced expiratory volume in first second);

Haemoglobin;

Questionnaire scores: GAD sum score, PHQ9 sum score, Mediterranean diet score, mental component score of SF36.
