# Supplementary material for: Predictors for one-year outcomes of cardiorespiratory fitness and cardiovascular risk factor control after cardiac rehabilitation in elderly patients: The EU-CaRE study
Source: PLoS One. 2021 Aug 5;16(8):e0255472. doi: 10.1371/journal.pone.0255472 (PMC8341663; doi:10.1371/journal.pone.0255472)
Supplement: Attachment — Submitted filename: Review Comments.docx [file pone.0255472.s004.docx]

**Comments**

This is a powerful paper which contributes to expand the knowledge about cardiovascular rehabilitation in elderly patients. In this paper, the authors analyzed the effect of cardiac rehabilitation in the enhancement of traditional cardiovascular risk factors – LDL-cholesterol, blood pressure, weight, and HbA1c in patients with DM - and improvement of functional capacity (peak oxygen consumption – peak VO2).

This paper is multicenter study (8 centers) with significant number of older individuals (n = 1633 participants) evaluated using gold standard methods (cardiopulmonary exercise testing) with high quality (“anaerobic threshold and a respiratory quotient (RQ) of >1.1.”) and with a high adherence rate in the cardiac rehabilitation programs (93%) and follow-up after 1 year (89%). The available of the lag time to start of the cardiac rehabilitation (CR) programme was a unique information of this study, which in elderly surgery patients seems to be the main determinant for improvement in functional capacity.

According to World Health Organization we are old at age 65 years. Because of the ageing population and medical progress, more and more old patients are addressed to cardiac rehabilitation centers. The biology of aging and the pathophysiology of cardiovascular disease (CVD) overlap, with the effect that CVD is endemic in the growing population of older adults. Moreover, CVD in older adults is usually complicated by age-related complexities, including multimorbidity, polypharmacy, frailty, and other intricacies that add to the risks of ambiguous symptoms, deconditioning, iatrogenesis, falls, disability, and other challenges. Importantly, participation in core components, such as exercise and nutritional interventions, can impact on both cardiac vascular disease and frailty. In this paper, understanding the behavior of important core components - blood pressure, LDL-cholesterol, weight and glycosylated hemoglobin - after rehabilitation will help guide and individualize cardiac rehabilitation in the elderly population.

Exercise-based CR programs have shown to be effective in improving function and quality of life, by reducing disability and age-related deconditioning and contributing favorably to improved health outcomes in an aged population. Despite these proven benefits, CR is often underused in this population and a great effort should be done to encourage them to attend these programs. This study can be instrumental to individualization of CR based on age, gender, weight, time to start the program and optimization of the result - improvement of functional capacity.

**Questions**

Line 92. “We aimed at including a total of 1760 patients equivalent of 220 patients from each site”.

How was the sample size calculated? In the design paper of the study - A EUropean study on effectiveness and sustainability of current Cardiac Rehabilitation programmes in the Elderly: Design of the EU-CaRE randomised controlled trial. European journal of preventive cardiology. 2016;23(2 suppl):27-40 – it is described the sample size of the Eu-Care RCT (randomised controlled trial) based on the improvement of peak O2 consumption, but not from the observational study.

**Suggestion of citation**

Kim KH, Jang YC, Song MK, Park HK, Choi IS, Han JY in the recent paper “Changes in Aerobic Capacity Over Time in Elderly Patients with Acute Myocardial Infarction During Cardiac Rehabilitation. Ann Rehabil Med. 2020 Feb;44(1):77-84. doi: 10.5535/arm.2020.44.1.77. Epub 2020 Feb 29. PMID: 32130841; PMCID: PMC7056327” found a similar no improvement in functional capacity in elderly patients in CR.

**Suggestion of limitation**

Almost 50% of CR participants are older adults (>65 years), many of whom are frail or deconditioned. We need to start looking for frailty in elderly patients entering CR programmes and become more familiar with the tools to recognize and evaluate the severity of this condition. Furthermore, we need to better understand whether exercise-based cardiac rehabilitation may change the course and the prognosis of frailty in cardiovascular patients. The benefits of CR are well-suited to counteract the deficits of frailty such as sarcopenia, inactivity, fatigue, cognitive decline, and depression.
