# Supplementary material for: Predictors for one-year outcomes of cardiorespiratory fitness and cardiovascular risk factor control after cardiac rehabilitation in elderly patients: The EU-CaRE study
Source: PLoS One. 2021 Aug 5;16(8):e0255472. doi: 10.1371/journal.pone.0255472 (PMC8341663; doi:10.1371/journal.pone.0255472)
Supplement: Attachment — Submitted filename: Responses to reviewers.docx [file pone.0255472.s005.docx]

**Point-by-point responses to reviewers’ comments**

We thank the reviewers for their constructive comments and hope we have addressed them accordingly. Please find below our responses to the reviewers’ comments in italic font.

Review Comments to the Author

Please use the space provided to explain your answers to the questions above. You may also include additional comments for the author, including concerns about dual publication, research ethics, or publication ethics. (Please upload your review as an attachment if it exceeds 20,000 characters)

Reviewer #1: Interesting paper.
Abstract; at firsr glance it remains unclear how patients were selected. Which were inclusion criteria?

*Detailed inclusion criteria have been published previously in: Prescott E, Meindersma EP, van der Velde AE, Gonzalez-Juanatey JR, Iliou MC, Ardissino D, Zoccai GB, Zeymer U, Prins LF, Van't Hof AW, Wilhelm M, de Kluiver EP. A EUropean study on effectiveness and sustainability of current Cardiac Rehabilitation programmes in the Elderly: Design of the EU-CaRE randomised controlled trial. Eur J Prev Cardiol. 2016 Oct;23(2 suppl):27-40. And*

*Prescott E, Mikkelsen N, Holdgaard A, Eser P, Marcin T, Wilhelm M, et al. Cardiac rehabilitation in the elderly patient in eight rehabilitation units in Western Europe: Baseline data from the EU-CaRE multicentre observational study. European journal of preventive cardiology. 2019;26(10):1052-63.*

*Additionally, we have amended the following sentence on line 97, page 6, as follows: “Briefly, elderly (65+) patients participating in CR of one of the eight European centres after coronary artery bypass grafting (CABG), percutaneous coronary intervention (PCI) or without revascularization as well as after percutaneous or surgical valve replacement (HVR) were included.”*

methods: definition of elderly should be added

*Elderly was defined as patients aged ≥65 years (please see sentence cited above).*

methods/results>a relevant difference between patients after CABG/cardiac surgery and after PCI is present
methods: it should be added how variavles were selected

*Selection of variables is described on page 8, line 138: “Robust linear mixed models were performed for change in peak VO2 [ml/min/kg] between T0 and T2 (function rlmer from package robustlmm version 2.3). The following factors were included as predictor variables: Age, sex and peak exercise capacity at T0, comorbidities, cardiovascular risk factors and characteristics of CR, namely, lag time of CR and CR duration and self-reported physical activity level during follow-up. Centre was entered into the model as a random factor. Further factors from a list of cardiovascular risk factors and comorbidities (provided in S 1 Table) by backward selection. Non-significant variables were eliminated from the model manually by individually removing the variable with the largest p-value until any insignificant (p>0.01) parameters were removed from the model. This was done to avoid removing non-significant variables which had a large effect on other significant variables.”*

methods: primary end point and secondary end points should be added

*Primary and secondary end points were the predictors for the following primary and secondary outcomes (page 7, line 130): “Predictors were sought for the primary outcome, changes in CRF (T2-T0), namely change in peak VO2 [ml/kg/min]. Predictors were also sought for secondary outcomes, namely for CVRF control at T1 and T2, including the achievement of target levels according to current guidelines [15] as follows: BPsys < 140 mmHg, LDL-C < 1.8 mmol/l, BMI < 30 kg/m2 (non-obesity) or lowering body weight by ≥5%, and HbA1c in diabetic patients < 53 mmol/mol.”*

methods: sample size calculation should be added

*The sample size calculation for the EU-CaRE observational study is stated on page 6, line 93. “We aimed at including a total of 1760 patients equivalent of 220 patients from each site..”*

Reviewer #2: This is a powerful paper which contributes to expand the knowledge about cardiovascular rehabilitation in elderly patients. In this paper, the authors analyzed the effect of cardiac rehabilitation in the enhancement of traditional cardiovascular risk factors – LDL-cholesterol, blood pressure, weight, and HbA1c in patients with DM - and improvement of functional capacity (peak oxygen consumption – peak VO2).

(Also see attached document with comments)

Reviewer #3: I commend the authors on this work and after having reviewed the manuscript have the following queries:

General comments:
Please verify first expansion of all abbreviations and ensure that is done only once

*Thank you for pointing this out, we have corrected some missing or duplicated abbreviations.*


Specific comments:
1. Did the sites of recruitment have an influence on your results? This could be a confounder

*This is a good comment, yes, sites were a confounder, because CR programmes differed in duration and intensity from each other. Further, patient inclusion time into the programmes differed as well, with some centres starting patients in their programmes within days after interventions (stationary programmes) and several weeks or months (ambulatory programmes). However, we wanted to find predictors with regard to patient characteristics, not centre characteristics, which is why we included centres as random factors. The influence of site has been addressed in the publication of the main study results: Prescott E, Eser P, Mikkelsen N, Holdgaard A, Marcin T, Wilhelm M, Gil CP, González-Juanatey JR, Moatemri F, Iliou MC, Schneider S, Schromm E, Zeymer U, Meindersma EP, Crocamo A, Ardissino D, Kolkman EK, Prins LF, van der Velde AE, Van't Hof AW, de Kluiver EP. Cardiac rehabilitation of elderly patients in eight rehabilitation units in western Europe: Outcome data from the EU-CaRE multi-centre observational study. Eur J Prev Cardiol. 2020 Nov;27(16):1716-1729.*

2. I understand the numbers of those not completing CR are small - but did you attempt to identify any factors within that group as well (if they have CPET values)?

*Unfortunately, none of the centres had CPET data from patients not undergoing CR.*

3. Were any functional test performed in the elderly? This could be used as surrogates among those in whom CPET was not possible

Indeed, 6-minute-walking tests were performed in patients who were too weak to perform CPETs. We have presented detailed results in:

*“A CPET was performed by 1534 patients, 48 performed a 6MWT and 51 patients did not have a baseline functional test available.” We have presented these results in detail in:*

*Prescott E, Eser P, Mikkelsen N, Holdgaard A, Marcin T, Wilhelm M, Gil CP, González-Juanatey JR, Moatemri F, Iliou MC, Schneider S, Schromm E, Zeymer U, Meindersma EP, Crocamo A, Ardissino D, Kolkman EK, Prins LF, van der Velde AE, Van't Hof AW, de Kluiver EP. Cardiac rehabilitation of elderly patients in eight rehabilitation units in western Europe: Outcome data from the EU-CaRE multi-centre observational study. Eur J Prev Cardiol. 2020 Nov;27(16):1716-1729.*
