# Supplementary material for: Predictors for one-year outcomes of cardiorespiratory fitness and cardiovascular risk factor control after cardiac rehabilitation in elderly patients: The EU-CaRE study
Source: PLoS One. 2021 Aug 5;16(8):e0255472. doi: 10.1371/journal.pone.0255472 (PMC8341663; doi:10.1371/journal.pone.0255472)
Supplement: Attachment — Submitted filename: Responses to Review Comments_2.docx [file pone.0255472.s006.docx]

**Responses to reviewer 2**

Thank you very much for your supportive comments. Please find below our responses to your comments and questions. We hope that we have addressed them according to your expectations.

**Questions**

Line 92. “We aimed at including a total of 1760 patients equivalent of 220 patients from each site”.

How was the sample size calculated? In the design paper of the study - A EUropean study on effectiveness and sustainability of current Cardiac Rehabilitation programmes in the Elderly: Design of the EU-CaRE randomised controlled trial. European journal of preventive cardiology. 2016;23(2 suppl):27-40 – it is described the sample size of the Eu-Care RCT (randomised controlled trial) based on the improvement of peak O2 consumption, but not from the observational study.

*The EU-CaRE study consisted of an observational study and an RCT. For the observational study, sample size was based on number of expected eldery patients seen each year and consisted of 220 patients per centre. In the methods section on p. 6 it reads:* We aimed at including a total of 1760 patients equivalent of 220 patients from each site. The CR program offered at each site has been described previously [12].

*Ref. 12 (see above) refers to the description of the CR program only, not to the sample size calculation.*

**Suggestion of citation**

Kim KH, Jang YC, Song MK, Park HK, Choi IS, Han JY in the recent paper “Changes in Aerobic Capacity Over Time in Elderly Patients with Acute Myocardial Infarction During Cardiac Rehabilitation. Ann Rehabil Med. 2020 Feb;44(1):77-84. doi: 10.5535/arm.2020.44.1.77. Epub 2020 Feb 29. PMID: 32130841; PMCID: PMC7056327” found a similar no improvement in functional capacity in elderly patients in CR.

*Thank you for pointing out this reference, we have included it in the Discussion section.*

**Suggestion of limitation**

Almost 50% of CR participants are older adults (>65 years), many of whom are frail or deconditioned. We need to start looking for frailty in elderly patients entering CR programmes and become more familiar with the tools to recognize and evaluate the severity of this condition. Furthermore, we need to better understand whether exercise-based cardiac rehabilitation may change the course and the prognosis of frailty in cardiovascular patients. The benefits of CR are well-suited to counteract the deficits of frailty such as sarcopenia, inactivity, fatigue, cognitive decline, and depression.

*We couldn’t agree more!*
